# Abstinence from heroin self-administration increases dendritic spine head diameter and the proportion of mushroom spines in rat prelimbic cortical neurons projecting to the paraventricular thalamus

**DOI:** 10.21203/rs.3.rs-10797923/v1

**Published:** 2026-09-04

**Authors:** Maya A. Nash, Giuseppe Giannotti

**Affiliations:** Washington State University; Washington State University

**Keywords:** heroin, prelimbic cortex, paraventricular thalamus, dendritic spines, abstinence, structural plasticity

## Abstract

Relapse to opioid use remains the principal obstacle to recovery, often driven by enduring neuroadaptations that sustain vulnerability across abstinence. We have previously shown that the projection from the rat prelimbic cortex (PL) to the paraventricular thalamus (PVT) is functionally potentiated following abstinence from heroin self-administration and is necessary for cued heroin seeking, yet whether this pathway undergoes structural remodeling remains unknown. Using an intersectional viral strategy to selectively label PL→PVT projection neurons, we reconstructed apical tuft dendritic spines in three dimensions from rats following 14 days of home-cage abstinence from heroin self-administration. We found that abstinence significantly increased spine head diameter (d_H_) and shifted the entire head-diameter distribution without altering overall spine density. Using an unsupervised model-based clustering to avoid arbitrary morphometric thresholds, we identified three distinct spine populations corresponding to classical thin, stubby, and mushroom categories. We found that abstinence reduced the density of thin-like spines and increased the density of mushroom-like spines. Notably, this accumulation of mature, mushroom-like spines positively correlated with the total amount of heroin consumed during self-administration. Overall, these findings identify the structural potentiation of excitatory inputs onto PL→PVT projection neurons as a fundamental component of the enduring circuit plasticity that sustains heroin seeking.

## Introduction

Opioid use disorder (OUD) is a chronic, relapsing disorder characterized by a persistent vulnerability to drug seeking and relapse, even after prolonged periods of abstinence. In abstinent individuals with OUD, exposure to drug-associated cues increases craving and impairs decision-making at abstinence durations ranging from one to twenty-four months (Wang et al. 2012), indicating that cue reactivity remains a durable feature of the disorder. In preclinical models, cue-induced opioid seeking progressively escalates across abstinence (Chow et al. 2025; Venniro et al. 2016). This time-dependent increase in seeking indicates that abstinence actively drives enduring neuroadaptations within the circuits regulating cue-driven behavior. Therefore, identifying the circuit-specific plasticity that emerges during abstinence is critical for understanding the neural mechanisms that sustain opioid relapse.

The paraventricular thalamus (PVT) is a key integrative node within neural circuits regulating motivated behavior and drug seeking in rodents (James and Dayas 2013; Kirouac 2015; Zhou and Zhu 2019; McGinty and Otis 2020; Iglesias and Flagel 2021) with converging evidence implicating thalamic circuitry in human substance use disorders (Huang et al. 2018). Work from our laboratory and others has established an important role for downstream PVT projections, particularly those targeting the nucleus accumbens (NAc), in opioid withdrawal and heroin seeking (Giannotti et al. 2021; Paniccia et al. 2024; Zhu et al. 2016; Keyes et al. 2020; Vollmer et al. 2022). In contrast, considerably less is known about the upstream afferents recruiting and regulating PVT activity that sustain opioid seeking. Among these afferents, the prelimbic cortex (PL) provides a major excitatory monosynaptic input to the PVT (Li et al. 2024; Li and Kirouac 2012; Kirouac 2024; Gabbott et al. 2005) and has emerged as an important regulator of cue-driven behaviors (Giannotti et al. 2018; Otis et al. 2017; Campus et al. 2019; Kuhn et al. 2022).

Dendritic spines are the principal sites of excitatory synaptic input, and their morphology is closely associated with synaptic strength. Spine head size scales with postsynaptic density area and AMPA receptor content, with larger heads generally representing stronger and more stable synapses (Kasai et al. 2003; Matsuo et al. 2008; Matsuzaki et al. 2001; Sala and Segal 2014). Consistent with this relationship, abstinence from cocaine self-administration increases spine head diameter (d_H_) in PL neurons projecting to the NAc core, together with increased GluA1 and GluA2 localization within enlarged spine heads (Siemsen et al. 2019). Similarly, heroin self-administration followed by extinction increases spine d_H_ in the same projection and is accompanied by increased GluA1 immunoreactivity in dendritic spine heads (Siemsen et al. 2023). However, whether similar structural remodeling occurs in other relapse relevant PL outputs remains unknown.

The PL→PVT pathway is a compelling candidate for such remodeling. We recently showed this pathway is necessary for cued heroin seeking and undergoes synaptic potentiation after 14 days of abstinence (Jensen et al. 2025), yet whether the PL neurons themselves are structurally remodeled is unknown. Therefore, here we sought to determine whether the synaptic potentiation of the PL→PVT pathway (Jensen et al. 2025) is accompanied by structural plasticity in the neurons that give rise to it. Using an intersectional viral strategy, we selectively labeled PL neurons projecting to the PVT and quantified apical tuft dendritic spine morphology after 14 days of home-cage abstinence from heroin self-administration. We found that abstinence increased dendritic spine d_H_ and shifted the spine population from thin-like toward mushroom-like spines, without changing spine density. These findings identify abstinence-induced structural remodeling within the PL→PVT projection neurons, extending our previous functional and synaptic observations (Jensen et al. 2025) and supporting this circuit as a site of adaptations contributing to heroin seeking.

## Materials and Methods

### Subjects

Age-matched male and female Wistar rats (postnatal day 55–60 on arrival; Envigo RMS Division) were used (n = 16; 8 males, 8 females). Four animals were excluded from the final dataset for absent viral expression in the PL (3 saline, 1 heroin). Rats were individually housed in standard ventilated cages in a climate- and humidity-controlled vivarium on a 12 h reverse dark/light cycle (lights off at 9:00 A.M.), with ad libitum access to standard chow and water. All experiments were conducted during the dark cycle (9:00 A.M. − 9:00 P.M.). All procedures were approved by the Washington State University Institutional Animal Care and Use Committee and conducted in an AAALAC-accredited facility.

### Intravenous surgery

Rats were anesthetized with ketamine/xylazine (80/7 mg/kg) prior to surgery, with ketamine boosters given as needed to maintain the surgical plane of anesthesia. An intravenous jugular catheter was implanted as described previously (Jensen et al. 2025). Briefly, the distal end of the silastic tubing was inserted into the right jugular vein, and the other end was passed subcutaneously over the shoulder to a 22-gauge cannula mounted on the animal’s back. Carprofen (5 mg/kg) and cefazolin (100 mg/kg) were administered to alleviate surgical pain and prevent infection, respectively, and taurolidine-citrate solution was used as the catheter lock to prevent occlusion. Rats were allowed to recover for at least one week before the beginning of self-administration training.

### Viral injections

Viral injections were performed during the same surgical session as jugular catheter implantation. The PVT was injected with 0.3 μL of AAVrg-hSyn-Cre-WPRE-hGH (titer 1.9 × 10^13^ vg/mL; Addgene viral prep #105553-AAVrg), and the PL was bilaterally injected with 0.5 μL/side of AAV5-hSyn-DIO-mCherry (titer 2.2 × 10^13^ vg/mL; Addgene viral prep #50459-AAV5). All viral injections were performed using coordinates from Paxinos and Watson (2014) relative to bregma. PL coordinates were anteroposterior (A/P) + 2.8 mm, mediolateral (M/L) ± 0.6 mm, and dorsoventral (D/V) − 3.8 mm. PVT coordinates were A/P − 2.8 mm, M/L − 2.41 mm at a 25° angle, and D/V − 5.74 mm. Viral injections were performed using a Nanoject III nanoliter injector (Drummond Scientific, Broomall, PA, USA) at a rate of 20 nL/s through a borosilicate glass tip. Following each infusion, the glass tip remained in place for 10 min to allow diffusion of the viral preparation.

### Heroin and saline self-administration

Following one week of recovery, rats underwent 12 days of heroin self-administration in standard operant chambers (Med Associates, St. Albans, VT, USA) housed within ventilated, sound-attenuated cubicles. Rats self-administered heroin (40 μg/50 μL/2.85 s) for 3 h/day under an escalating fixed-ratio schedule consisting of FR1 for 7 days, FR2 for 2 days, and FR4 for the final 3 days, consistent with our previous studies (Giannotti et al. 2021; Jensen et al. 2025). Active lever presses resulted in intravenous heroin delivery accompanied by a 5 s cue consisting of a light above the active lever and a 3.5 kHz tone. Each infusion was followed by a 20 s timeout period during which the lever was retracted and heroin was unavailable. Inactive lever presses were recorded but had no programmed consequences. Saline control animals underwent identical self-administration procedures with contingent intravenous infusions of sterile 0.9% saline. Diamorphine HCl was obtained through the NIDA Drug Supply Program and dissolved in sterile 0.9% saline, at a final concentration of 0.8 mg/mL. Catheter patency was assessed as needed using methohexital sodium (10 mg/mL), which produces a brief loss of righting reflex.

### Immunohistochemistry

Following the final self-administration session, rats underwent 14 d of home-cage abstinence, matching the withdrawal interval at which PL→PVT synaptic potentiation and heroin seeking were observed previously (Jensen et al. 2025). On abstinence day 14, rats were transcardially perfused with 0.9% saline followed by 10% formalin. Brains were extracted, post-fixed for 3 h in 10% formalin, cryoprotected in 20% sucrose for at least 48 h, flash-frozen, and stored at − 80°C until sectioning. Coronal sections (40 μm) containing the PL were cut on a Leica CM1860 cryostat (Leica Microsystems) and collected in chilled 0.1 M phosphate-buffered saline (PBS) containing 1% sodium azide. Free-floating sections were incubated for 1 h in 2% normal donkey serum in PBS with 0.3% Triton X-100, then incubated overnight at room temperature with a rabbit anti-mCherry primary antibody (1:1,000; LSBio, catalog #LS-C204825–100; RRID:AB_2716246). After three 5 min washes in PBS, sections were incubated for 2 h in PBS-T with 2% NDS and goat anti-rabbit Alexa Fluor 594 (1:1,000; Abcam, catalog #ab150080; RRID:AB_2650602). Sections were mounted onto slides and coverslipped with Fluoromount-G (Electron Microscopy Sciences) before imaging.

### Confocal imaging

Imaging and reconstruction procedures followed those described previously (Siemsen et al. 2023; Siemsen et al. 2019). Confocal z-stacks were acquired on a Leica SP8 laser-scanning confocal microscope (Leica Microsystems) using a 63× oil-immersion objective (1.4 NA) with 4x digital zoom, a 400 Hz acquisition rate, and a 0.1 μm z-step size. Images were collected at a frame size of 1024 × 1024 pixels with 12-bit resolution and 4 frame averages. mCherry fluorescence was excited using a 598 nm laser line, and emitted photons were collected using high-sensitivity hybrid detectors (HyD). Laser power, gain, pinhole, and optical section thickness were empirically determined prior to imaging and held constant across all samples. Images were acquired using sub-Airy unit pinhole sizes to optimize resolution for three-dimensional reconstruction. Segments of approximately 50 μm were imaged, and two to four segments were sampled per animal. Image stacks were deconvolved using a custom three-dimensional Richardson–Lucy algorithm (Richards and Wolf 1959) implemented in Python (NumPy/CuPy) prior to reconstruction. A theoretical confocal point-spread function (psf) was computed for each stack with the psf libraries Gohlke and Holub, which implement the vectorial diffraction model of Richards and Wolf (1959).

### Three-dimensional reconstruction and spine morphology

Deconvolved z-stacks were imported into Imaris software (Bitplane, Oxford Instruments) for three-dimensional reconstruction, as previously described (Siemsen et al. 2023; Siemsen et al. 2019). Dendritic segments were first reconstructed using the Surface module to generate a three-dimensional mask of the labeled dendritic shaft. The Filament module was then used to semi-automatically trace dendritic segments and detect associated dendritic spines. Detection parameters were adjusted empirically and maintained consistently across all images. Spine reconstructions were manually inspected and corrected when necessary to ensure accurate representation of spine morphology. Spine density was calculated as the number of spines normalized to the length of the reconstructed dendritic segment (spines/μm). Spine d_H_ and morphological classification were extracted from the reconstructed spine objects.

### Spine classification

Spines were classified using model-based clustering (Scrucca et al. 2016; Fraley and Raftery 2002) as previously applied to human cortical dendritic spines (Luengo-Sanchez et al. 2018), rather than fixed morphometric thresholds traditionally used to define spine categories (Rodriguez et al. 2008). In brief, spine head diameter, neck diameter, and spine length were standardized and entered into a Gaussian mixture model (GMM) with full covariance structure, so that spines were clustered in the three-dimensional space defined by these measurements, fitted by expectation-maximization with 10 random restarts using the Python *scikit-learn* (Pedregosa et al. 2011). Clustering was performed blind to experimental group. The number of clusters was set at the elbow of the Bayesian information criterion (BIC) curve (k = 3). Rather than being assigned exclusively to one cluster, each spine contributed to all three in proportion to its posterior probability of membership. After fitting, clusters were labelled by reference to the classical spine categories of Peters and Kaiserman-Abramof (1970), with the cluster lacking a measurable neck designated stubby-like and, of the remaining two, the smaller-headed thin-like and the larger-headed mushroom-like.

### Statistical analysis

All statistical analyses were performed using GraphPad Prism (version 10.4.2; GraphPad Software; RRID:SCR_002798). Lever presses were analyzed separately for each group (Heroin and Saline) using two-way repeated-measures (RM) ANOVA with Day and Lever type (active vs inactive) as within-subject factors. Infusions were analyzed across groups using two-way RM ANOVA with Day as a within-subject factor and Self-Administration Type as a between-subject factor. Spine head diameter, spine voxel intensity, and dendritic spine density were analyzed using nested t-tests, with Self-Administration Type as the fixed factor and animal as the nesting variable. The dH frequency distribution (0.2 μm bins) and the cluster densities were analyzed using two-way RM ANOVA with Bin or Cluster as within-subject factors and Self-Administration Type as a between-subject factor, followed by Bonferroni-corrected multiple comparisons. The cumulative distribution of all reconstructed spines was compared using a Kolmogorov–Smirnov test in Python using *SciPy* (Virtanen et al. 2020), and relationships between cluster density and total heroin intake were assessed using Pearson correlations. Statistical significance is denoted in the figures (*p < 0.05, **p < 0.01, ***p < 0.001), with exact p values reported in the Results and biological replicates (n) in the figure legends, including the number of animals, dendritic segments, and spines. Significance was defined as alpha < 0.05, and data are graphed as mean ± SEM, with individual data points shown where applicable.

## Results

### Heroin and Saline self-administration training

To label PL neurons projecting to the PVT, we used a combinatorial viral approach. A retrograde virus carrying a Cre-recombinase (AAVrg-hSyn-Cre) was infused into the PVT and a Cre-dependent mCherry (AAV5-hSyn-DIO-mCherry) was injected bilaterally into the PL (Fig. 1a). Following a week of recovery, rats underwent 12 d of heroin or saline self-administration under increasing fixed ratio (FR) requirements (Fig. 1b, timeline). Heroin rats acquired self-administration over 12 d (Fig. 1c; two-way RM ANOVA; Day: F_(11,66)_ = 36.01, p < 0.0001; Lever type: F_(1,6)_ = 221.5, p < 0.0001; Day by Lever type interaction: F_(11,66)_ = 59.41, p < 0.0001). In contrast, saline rats did not reliably discriminate between the active and inactive levers (Fig. 1d; two-way RM ANOVA; Day: F(11,44) = 2.54, p = 0.0141; Lever type: F(1,4) = 5.41, p = 0.0806; Day by Lever type interaction: F_(11,44)_ = 1.99, p = 0.0529). Heroin animals escalated their intake across training while saline infusions declined, resulting in a significant Day by Treatment interaction (Fig. 1e; two-way RM ANOVA; Day: F_(11,110)_ = 2.59, p = 0.0058; Treatment: F_(1,10)_ = 2.87, p = 0.1212; Day by Treatment interaction: F_(11,110)_ = 6.46, p < 0.0001).

### Abstinence enlarges dendritic spine d_H_ on PL→PVT neurons

Following 14 d of abstinence, mCherry expression was examined in every animal and was restricted to the prelimbic cortex in both groups, confirming selective labeling of PL neurons projecting to the PVT (Fig. 2a, Saline; Fig. 2b, Heroin). Apical tuft dendrites of retrogradely identified PL→PVT projection neurons were then imaged and reconstructed in three dimensions (1,317 spines from 35 dendritic segments; Fig. 2c; top, Saline; bottom, Heroin). Spine dH was larger in heroin rats than in saline controls (Fig. 2d; nested t test; t_(10)_ = 4.78, p = 0.0007). Because Imaris reconstructs spines from a fluorescence threshold, brighter labeling could in principle yield larger reconstructed surfaces. To rule out this possibility, we compared mean voxel intensity within the reconstructed spines, which did not differ between groups (Fig. 2e; nested t test; t_(10)_ = 1.01, p = 0.3383), indicating that the enlargement cannot be attributed to a difference in labeling intensity. Moreover, dendritic spine density was unchanged (Fig. 2f; nested t test; t_(10)_ = 0.36, p = 0.7233), indicating that abstinence acts on existing spines rather than adding or eliminating them. Notably, the enlargement reflected a shift of the entire spine population. Analyses of the d_H_ frequency distribution revealed a difference between groups (Fig. 2g; two-way RM ANOVA; Bin: F(5,50) = 89.83, p < 0.0001; Bin by treatment interaction: F(5,50) = 14.66, p < 0.0001), and Bonferroni's post hoc comparisons showed that heroin rats had fewer spines in the 0.2–0.4 μm bin (p < 0.0001) and more spines in the 0.6–0.8 μm bin (p = 0.0021). The same shift was apparent in the cumulative distribution of all reconstructed spines (Fig. 2h; Kolmogorov–Smirnov test; D = 0.287, p < 0.0001). Together, these data indicate that abstinence from heroin self-administration shifts the spine population on PL→PVT neurons toward larger heads without altering spine number, a structural signature of strengthened excitatory input onto these neurons.

### Heroin shifts the spine population from thin-like to mushroom-like spines

Assigning spines to discrete types conventionally requires fixed cut-offs, so the resulting proportions depend on where those boundaries are drawn. We therefore clustered spines using an unsupervised Gaussian mixture model (GMM) fitted to head diameter, neck diameter, and spine length, blind to treatment group (Fig. 3a and b). The number of clusters was determined by the elbow of the Bayesian information criterion (BIC) curve (k = 3). The resulting components corresponded closely to the classical spine categories and comprised a thin-like component with small heads and narrow necks, a stubby-like component defined by the absence of a measurable neck, and a mushroom-like component with larger heads and wider necks. We next asked whether heroin altered the abundance of these populations. We found that cluster density differed between groups (Fig. 3c; two-way RM ANOVA; Cluster F_(2,20)_ = 66.73, p < 0.0001; Cluster by treatment interaction F_(2,20)_ = 14.24, p = 0.0001). Bonferroni's post hoc comparisons revealed that abstinence from heroin self-administration decreased the density of thin-like spines (p = 0.0003) and increased density of mushroom-like spines (p = 0.038) compared to Saline controls, while stubby-like density was unchanged (p > 0.99). Importantly, because the clusters were defined without reference to treatment group, this redistribution is a property of the data rather than a consequence of classification criteria. We next examined whether this remodeling varied with the amount of heroin consumed during the heroin self-administration training. Cluster densities and total heroin intake were first compared in a correlation matrix (Fig. 3d), and the individual relationships were then decomposed by simple linear regression. Thin-like (Fig. 3e; r = 0.42; p = 0.35) and stubby-like density (Fig. 3f; r = − 0.17; p = 0.71) were unrelated to intake, whereas mushroom-like density increased with total heroin intake (Fig. 3g; r = 0.78; p = 0.039). Together, these data indicate that abstinence from heroin self-administration shifts the spine population on PL→PVT neurons from thin-like toward larger, more stable mushroom-like spines, consistent with a strengthening of excitatory input onto this projection, and that this accumulation of mushroom-like spines increases with the amount of heroin consumed.

## Discussion

In the present study we found that 14 days of abstinence from heroin self-administration enlarged dendritic spine d_H_ on PL→PVT projection neurons without altering overall spine density. The increase in d_H_ was accompanied by a redistribution from thin-like toward mushroom-like spines, consistent with remodeling of existing spines rather than a net change in spine number. Together with our previous work showing increased synaptic strength at PL→PVT synapses following 14 days of abstinence from heroin self-administration (Jensen et al. 2025), these findings identify the PL→PVT pathway as a site of enduring abstinence-induced structural and functional plasticity following abstinence from self-administered heroin.

Spine head enlargement is closely associated with functional synaptic potentiation. At individual dendritic spines, increase in head size correlates with potentiation of AMPA receptor-mediated currents, while persistent spine enlargement accompanies sustained increases in excitatory postsynaptic potentials (Matsuzaki et al. 2001; Matsuzaki et al. 2004). Moreover, *in vivo* two-photon glutamate uncaging combined with whole-cell recording in neocortex of mice showed that AMPA receptor-mediated currents evoked at individual spines scale with spine-head volume (Noguchi et al. 2011). Within this framework, the increase in spine head diameter observed in the present study could reflect a potentiated excitatory drive onto PL neurons projecting to the PVT. By simultaneously amplifying the excitatory drive onto these projection neurons and potentiating their downstream thalamic synapses (Jensen et al. 2025), this coordinated plasticity may create a hyper-responsive circuit primed to drive cue-induced heroin seeking following abstinence (Jensen et al. 2025).

The structural remodeling of PL→PVT neurons observed here resembles adaptations reported in the PL neurons projecting to the Nucleus Accumbens (NAc), suggesting that enlargement of apical dendritic spines may be a recurring feature of withdrawal-induced plasticity across prelimbic output pathways. Following heroin self-administration and extinction training, PL→NAc core neurons exhibit enlarged apical-tuft spine heads accompanied by increased GluA1 immunoreactivity within those spines (Siemsen et al. 2023). Moreover, abstinence from cocaine self-administration produces a temporally dynamic pattern in the PL→NAc core projection neurons, with spine d_H_ initially reduced during early withdrawal but increased after 7 days of abstinence, when GluA1 and GluA2 immunoreactivity is selectively elevated in larger spine heads (Siemsen et al. 2019). Importantly, our work extends those morphological changes to the PL→PVT pathway by demonstrating a comparable increase in spine d_H_ after 14 days of abstinence from heroin self-administration. Together, these studies suggest that increased PL spine d_H_ may represent a recurring structural adaptation across cortical output populations following drug exposure, although its emergence and associated changes in spine density and AMPA receptor content likely depend on the drug, duration and condition of withdrawal, and downstream projection target (Siemsen et al. 2023; Siemsen et al. 2019).

Beyond this structural convergence, withdrawal-induced synaptic adaptations within the PL neurons following abstinence show functional heterogeneity depending on projection target, neuronal identity, behavioral context and sex. It has been shown that 7 days of abstinence from heroin self-administration increases intrinsic excitability in both D1- and D2-expressing PL→NAc neurons while increasing postsynaptic strength selectively in the D1-expressing population (Kokane et al. 2023). Notably, these adaptations are normalized following cue-induced heroin seeking (Kokane et al. 2023). In contrast, 14 days of abstinence from heroin self-administration reduces spontaneous firing and excitatory synaptic drive in PL→VTA neurons in mice (Wang et al. 2025). Consistent with this functional heterogeneity, adaptations observed in PL pyramidal neurons following 14–21 days of forced abstinence from remifentanil self-administration varied with sex and the duration of prior drug exposure. After 10–16 remifentanil self-administration sessions, prelimbic pyramidal neurons in female mice required greater current injection to fire action potentials (increased rheobase) and showed a reduced frequency of AMPAR-mediated miniature excitatory currents, whereas neurons in male mice fired action potentials at lower current (decreased rheobase) (Anderson et al. 2021). Because in all those studies dendritic spine morphology was not examined, it remains unknown whether these functional adaptations are accompanied by corresponding changes in spine d_H_ or other forms of structural remodeling.

Within the PL→PVT pathway itself, re-exposure to a withdrawal-paired context increases action potential firing and reduces the afterhyperpolarization in morphine-dependent mice (Yu et al. 2021). Although Yu and colleagues did not determine whether abstinence alone produces persistent hyperexcitability in PL→PVT neurons, nor investigated structural plasticity, their findings demonstrate that an opioid-associated context can enhance the excitability of this projection. Importantly, the behavioral relevance of the PL→PVT recruitment is supported by our recent work showing that chemogenetic inhibition or optogenetic synaptic depotentiation of this pathway attenuates cue-induced heroin seeking following abstinence from heroin self-administration (Jensen et al. 2025). Together with the persistent structural remodeling observed here, these findings support a model in which heroin abstinence increases activity within the PL→PVT pathway for subsequent recruitment by drug-associated stimuli.

Beyond the increase in spine dH, the redistribution of spine morphologies observed in the present study provides further insight into the nature of this structural adaptation. Small, thin-like spines are generally more dynamic and typically support weaker excitatory synapses, whereas large, mushroom-like spines are more stable, contain more AMPA receptors, and support stronger synaptic connections (Bourne and Harris 2007; Kasai et al. 2003). Importantly, the reduction we observed in thin-like spine density was balanced by an increase in mushroom-like spine density, resulting in a shift in morphological states without a net change in spine number. Thus, heroin abstinence remodels the synaptic population on PL→PVT neurons toward larger and potentially more stable excitatory connections. The absence of change in overall spine density differs from the reduced apical spine density reported in PL→NAc core neurons following either heroin self-administration and extinction training or forced abstinence from cocaine self-administration (Siemsen et al. 2023; Siemsen et al. 2019), suggesting that changes in spine number may depend on projection target, drug, and withdrawal condition. Together, the increase in spine d_H_ and redistribution toward mushroom-like spines 14 days after the final heroin exposure are consistent with persistent structural stabilization and potentiation of excitatory synapses onto PL→PVT neurons (Bozdagi et al. 2010; Matsuzaki et al. 2001; Yang et al. 2008).

It is important to note that the PL→PVT pathway is not engaged uniformly across all forms of seeking. Its contribution varies with the stimulus that triggers behavior, type of reward, and with the physiological state of the animal. Inhibition of this pathway reduces cue-induced heroin seeking after abstinence (Jensen et al. 2025), cue- and context-induced cocaine seeking (Giannotti et al. 2018), and cue-induced reinstatement of cocaine seeking in sign-tracking rats (Kuhn et al. 2022). In contrast, optogenetic stimulation of this pathway does not alter renewal of conditioned responding for sucrose-predictive cues (Brown and Chaudhri 2023), and chemogenetic manipulation of this pathway has opposite effects depending on phenotype, with stimulation decreasing the incentive value of reward cues in sign-trackers and inhibition increasing it in goal-trackers (Campus et al. 2019). Moreover, chemogenetic activation or inhibition of corticothalamic projections does not alter the augmented heroin seeking produced by chronic food restriction, whereas activation of PVT projections to the nucleus accumbens shell attenuates it (Chisholm et al. 2021). Importantly, the remodeling described here was measured in sated, drug-free animals, under the same heroin self-administration and abstinence conditions in which this projection is required for cue-induced heroin seeking (Jensen et al. 2025). Because physiological state can determine which PVT circuits control seeking, it may equally shape the structural adaptations within them, and whether the remodeling we describe is preserved, absent, or differently expressed under a homeostatic challenge such as chronic food restriction remains to be tested.

Finally, the identity of the excitatory afferents associated with the enlarged spines on PL→PVT neurons remains unknown. The basolateral amygdala (BLA) represents one plausible candidate. It sends dense glutamatergic projections to the PL (Bacon et al. 1996; Hoover and Vertes 2007; McDonald 1991) that are recruited during cue- and context-driven behavior (Anderson and Petrovich 2018; Burgos-Robles et al. 2017; Sotres-Bayon et al. 2012). It has been shown that optogenetic inhibition of BLA terminals in the PL attenuates cue-induced reinstatement of cocaine seeking (Stefanik and Kalivas 2013). Moreover, pharmacological inactivation of the BLA alters PL neuronal firing during the acquisition and extinction of opioid reward memory (Sun and Laviolette 2012) and functional disconnection of BLA from PL signaling prevents the acquisition of this memory (Rosen et al. 2016). Together these findings implicate BLA→PL signaling in cue-directed behaviors and in associative learning. However, none of these studies identified the postsynaptic PL populations targeted by BLA afferents or tested whether opioid abstinence strengthens BLA synapses onto PL→PVT neurons. Thus, determining whether heroin abstinence selectively remodels BLA or other glutamatergic afferents onto PL→PVT neurons therefore remains an important direction for future studies.

In conclusion, in the present study we show that 14 days of abstinence from heroin self-administration increased spine d_H_ and shifted the spine population toward larger, more stable morphologies on PL→PVT neurons without altering overall spine density. Together with our previous evidence of potentiated PL→PVT output synapses and reduced cue-induced heroin seeking following inhibition of this pathway (Jensen et al. 2025), these findings identify the PL→PVT circuit as a site of persistent structural and synaptic plasticity across its input and output components. Future studies should identify the afferents that innervate PL→PVT projection neurons and determine how the remodeling described here contributes to cued heroin seeking.

## Figures and Tables

**Figure 1:**
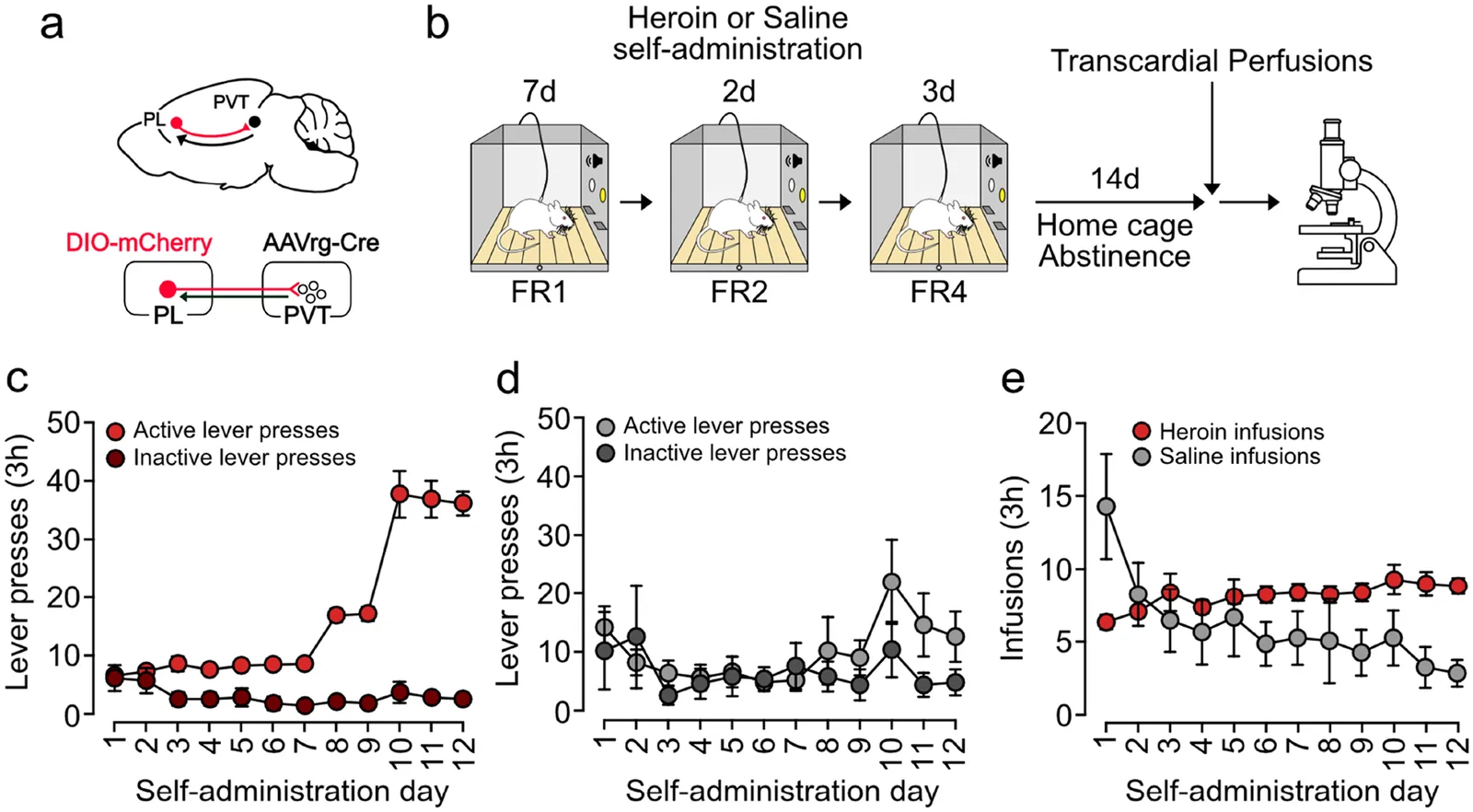
Intersectional labeling of PL→PVT projection neurons and heroin self-administration. **a)** Schematic of the viral strategy. An AAVrg expressing Cre was injected into the PVT and a Cre-dependent mCherry was bilaterally injected into the PL, restricting mCherry expression to PL neurons projecting to the PVT. **b)** Experimental timeline for heroin or saline self-administration, home-cage abstinence, and transcardial perfusion. **c)** Active and inactive lever presses during self-administration in heroin rats (n = 7) and **d)** saline rats (n = 5). **e)** Heroin and saline infusions earned per session. Data are shown as mean ± SEM

**Figure 2:**
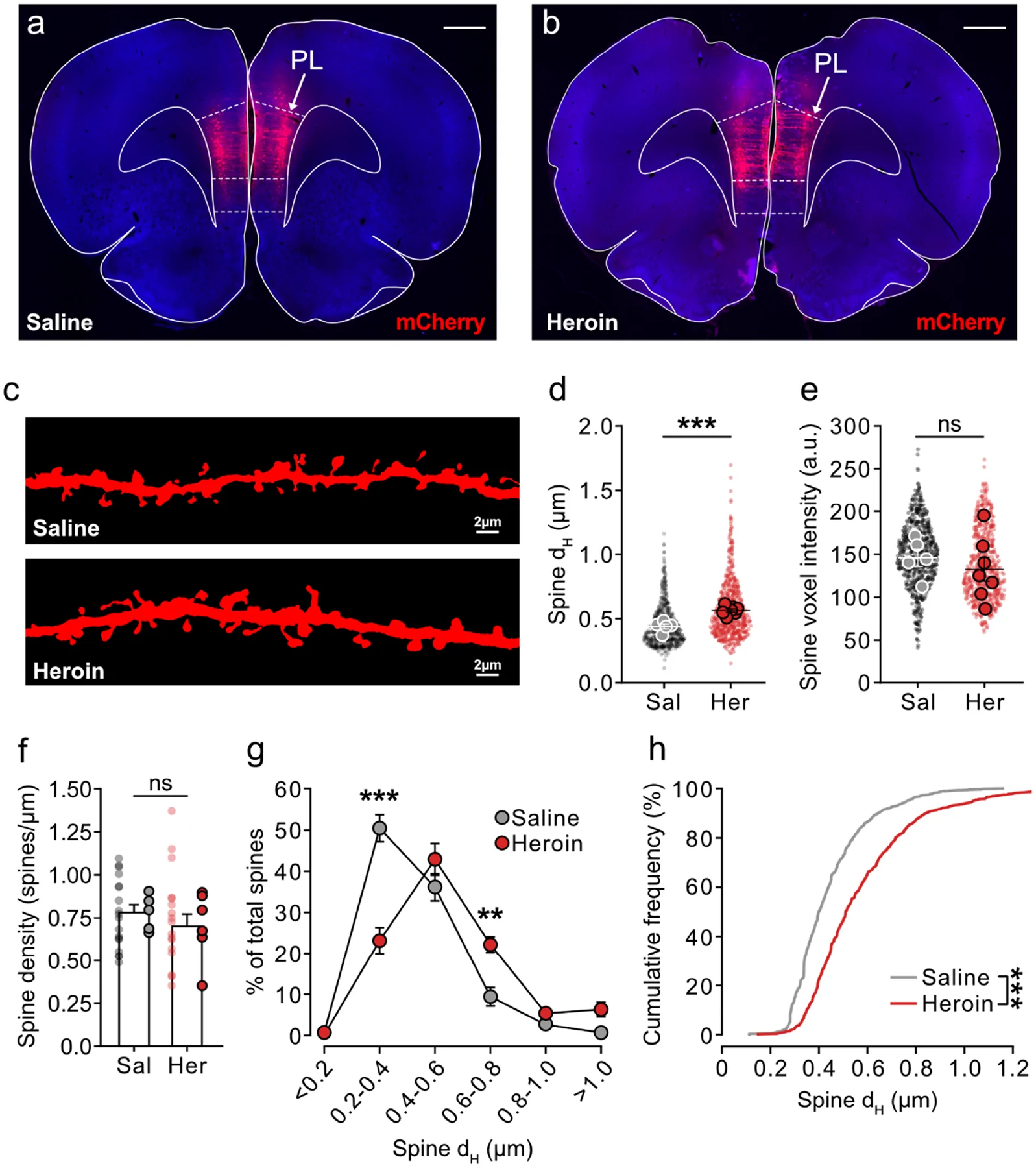
Abstinence from heroin self-administration enlarges dendritic spine heads on PL→PVT neurons. Representative wide-field images of mCherry expression (red) in the PL of a saline (**a**) and a heroin (**b**) rat. Empty channel autofluorescence (450 nm, blue) shows the outline of the section. Scale bars = 1 mm. **c)** Representative images of apical tuft dendritic segments from mCherry-labeled PL→PVT neurons in a saline (top) and a heroin (bottom) rat. Scale bars = 2 μm. **d)** Spine head diameter (d_H_) was increased in heroin rats. Small symbols are individual spines, and large symbols are animal means (saline, n = 693 spines/5 rats; heroin, n = 624 spines/7 rats). **e)** Mean voxel intensity within the reconstructed spines did not differ between groups. **f)** Spine density did not differ between groups. Small symbols are individual dendritic segments, and large symbols are animal means. **g)** Frequency distribution of spine d_H_, expressed as the percentage of each animal's spines in each 0.2 μm bin. **h)** Cumulative frequency distribution of spine d_H_ across all reconstructed spines. Data are shown as mean ± SEM and individual data points. ns not significant, ** p < 0.01, *** p < 0.001

**Figure 3:**
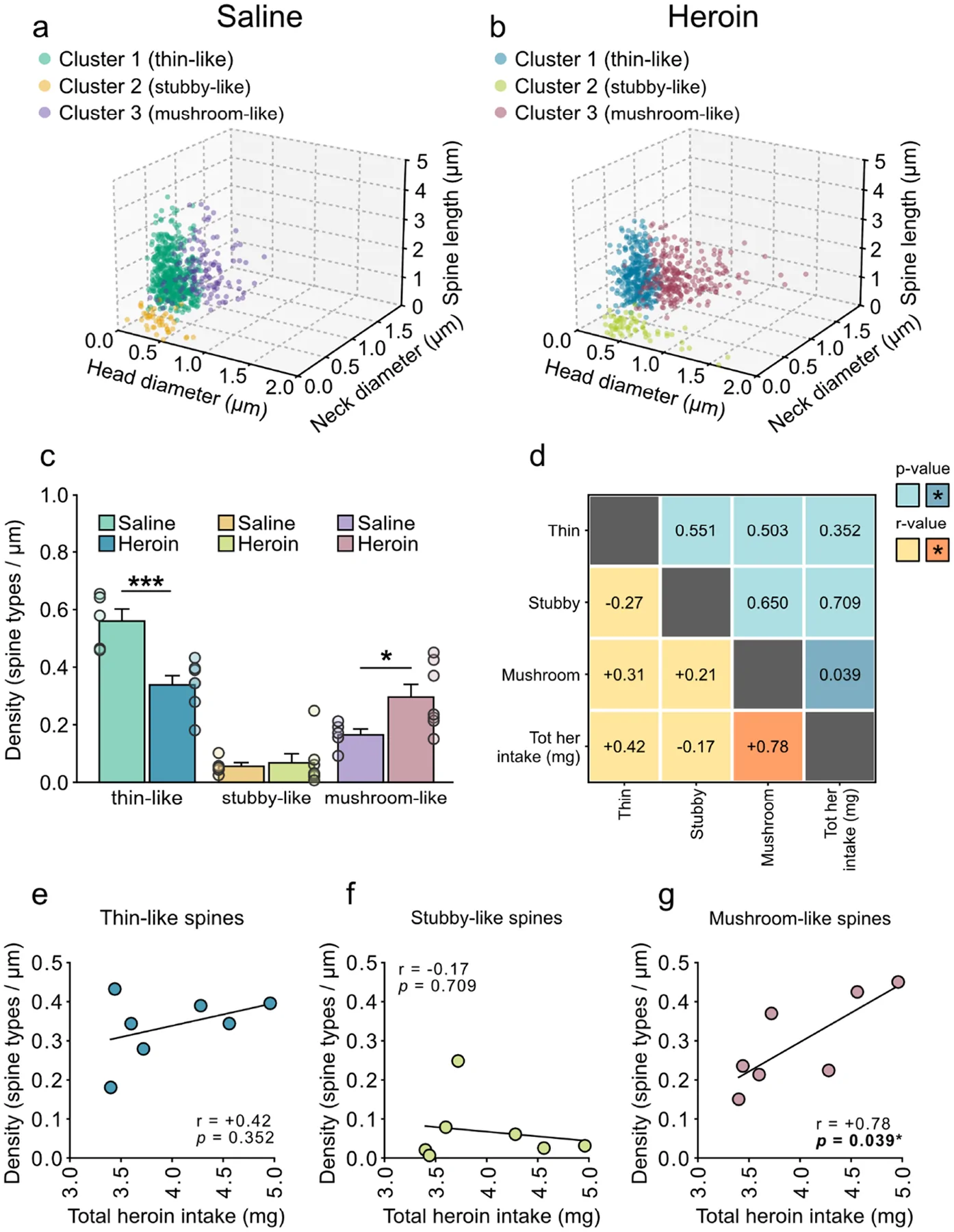
Threshold-free clustering reveals a redistribution of spine morphology after heroin abstinence. Gaussian mixture model (GMM) fitted to spine head diameter, neck diameter, and spine length in saline (**a**) and heroin (**b**) rats. Each point is one spine, colored by cluster assignment. **c)** Density of each spine cluster. Symbols are individual rats. **d)** Correlation matrix of cluster densities and total heroin intake in heroin rats, with Pearson r below the diagonal and the corresponding *p* value above it. Density of thin-like (**e**), stubby-like (**f**), and mushroom-like (**g**) spines against total heroin intake. Each symbol is one rat. Data are shown as mean ± SEM and individual data points. * p < 0.05, *** p < 0.001

## Data Availability

The datasets generated and analyzed during the current study are not publicly available but are available from the corresponding author on reasonable request.
